# Cochlear implantation in children with congenital inner ear anomalies: challenges and outcomes

**DOI:** 10.1007/s00405-025-09825-8

**Published:** 2025-11-18

**Authors:** Milan Urik, D. Hosnova, V. Kruntorad, Jan Sima

**Affiliations:** 1 Department of Paediatric Otorhinolaryngology, University Hospital Brno, Cernopolni 9, 61300 Brno, Czech Republic; 2 Faculty of Medicine, Masaryk University, Kamenice 753/5, 62500, Brno, Czech Republic

**Keywords:** Cochlear implant, Inner ear malformation, Cochlear nerve

## Abstract

**Purpose:**

Cochlear implantation (CI) is the state-of-the-art treatment option for sensorineural hearing loss condition including patients with congenital inner ear malformations. Recently our centre started treating children with CI who were diagnosed with inner ear malformations. Objectives of this retrospective study are to create three-dimensional (3D) model of malformed inner ears, identification of cochlear nerve bundle from pre-operative images, visualize electrode placement inside the cochlear portion from post-operative images, and evaluate hearing benefits post-operatively received from CI.

**Methods:**

Slicer software was used to 3D segment the inner ear and the electrode from the pre-, and post of CT scans. Using the same software, cross-section of internal auditory canal (IAC) was navigated to visualize the presence or absence of cochlear nerve. Speech intelligibility rating (SIR) and pure tone average (PTA) thresholds were evaluated for the benefits received from CI.

**Results:**

Our database showed a total of 12 children radiologically diagnosed with inner ear malformation and out of which 19 ears were treated with MED-EL CI devices. Enlarged vestibular aqueduct (EVA, incomplete partition (IP) types I, and II, cochlear hypoplasia (CH), cochlear aperture stenosis, common cavity (CA) were the different malformation types found in this cohort. Intra-operative gusher was observed in EVA and IP type II malformation types. Higher SIR scores and lower PTA thresholds were seen for less severe malformation types and vice-versa.

**Conclusions:**

Severity of malformation have a negative effect on the hearing outcomes with CI. MRI is an important tool in the identification of cochlear nerve bundle in subjects diagnosed with malformed inner ear anatomies.

## Introduction

Cochlear implant (CI) is the *state-of-the-art* treatment option for restoring hearing in sensorineural hearing loss condition including subjects with inner ear malformations [1]. The aim of the CI is to provide electrical stimulation equivalent to the received audio signal to surviving neuronal cell bodies inside the cochlea through the placement of electrode [2]. The signal is further transferred to the next level of auditory pathway to reach the auditory cortex where the electrical signal is perceived as meaningful sound [3]. An optimal placement of CI electrode inside the cochlea matching the cochlear size and anatomy is crucial in providing the electrical stimulation effectively in CI recipients [4].

A good outcome expectation with CI in subjects with normal inner ear anatomy is well reported in the literature, however only fewer reports on CI outcome in subjects with inner ear malformation is available in literature [5]. Ozkan et al. has reported better hearing outcomes with CI in subjects with less degree of severity of inner ear malformation and vice-versa, but nothing mentioned about the effect of electrode placement on the hearing outcomes [6]. Inner ear conditions like cochlear aperture stenosis and questionable auditory nerve even though with a normal development of the cochlear portion can lower the benefit of CI [6]. A good understanding on the anatomy of inner ear is important for planning the CI surgery especially with the placement of intra-cochlear electrode.

Incidence of inner ear malformation varies across the geographical location with 27% reported from USA [7], 84.6% in infants younger than 1 year of age from Japan [8], 30.69% from China [9], 15–20% from India [10], 17% from Poland [11], and 20% from Turkey [12]. Our centre has a good experience implanting CI in children with normal inner ear anatomy resulting in good hearing outcomes with CI. Recently we started treating children diagnosed with congenital inner ear malformation with CI and the initial hearing outcomes observed were convincing. This motivated us to retrospectively study all cases diagnosed with inner ear malformation in terms of three-dimensional (3D) model creation of inner ear and identification of cochlear nerve bundle from pre-operative images, electrode placement inside the cochlear portion from post-operative images, and hearing benefits post-operatively received from CI.

## Materials and methods

**Fig. 1:**
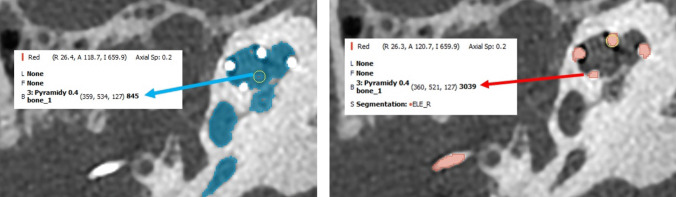
Example of greyscale thresholding when using CT images: membranous labyrinth (dark= labyrinth; 0 and 1000); electrode (bright= bone and metal; > 2500)

This study was approved by the ethics committee of the university hospital (01–160725/EK) to access both pre-, and post-operative images of all CI subjects implanted in our centre between 2019 and 2023 and radiologically diagnosed with inner ear malformation. Both computer tomography (CT) images and magnetic resonance images (MRI) of the identified CI subjects with inner ear malformation types were loaded into 3D slicer freeware (3D Slicer, https://www.slicer.org/; version 5.6.0, Massachusetts, USA) followed by segmentation of inner ear from pre-op images and electrode from post-op images. Greyscale threshold was set tight to differentiate inner ear structures filled with liquid and electrode contacts. Figure 1 shows an example of greyscale thresholding when using CT images; greyscale thresholding of membranous labyrinth (dark = labyrinth; 0 and 1000); greyscale thresholding of electrode (bright = bone and metal; > 2500). Classification of malformation types were made following the three-step approach recently proposed by Dhanasingh et al. [13]. Cross-section of internal auditory canal in MRI to check for the presence of cochlear nerve bundle was performed following the procedure described by Assiri et al. [14]. Pure tone average (PTA) at 6 months post-op was performed to check the hearing threshold with CI. Speech intelligibility rating (SIR) test was performed either at 1- or 3-year post-op time point as described by Cox et al. to evaluate the efficacy of CI treatment with a scale of 0–5 (0: no intelligible speech; 5: speech is understandable) [15].

## Results

### Demographics

**Fig. 2:**
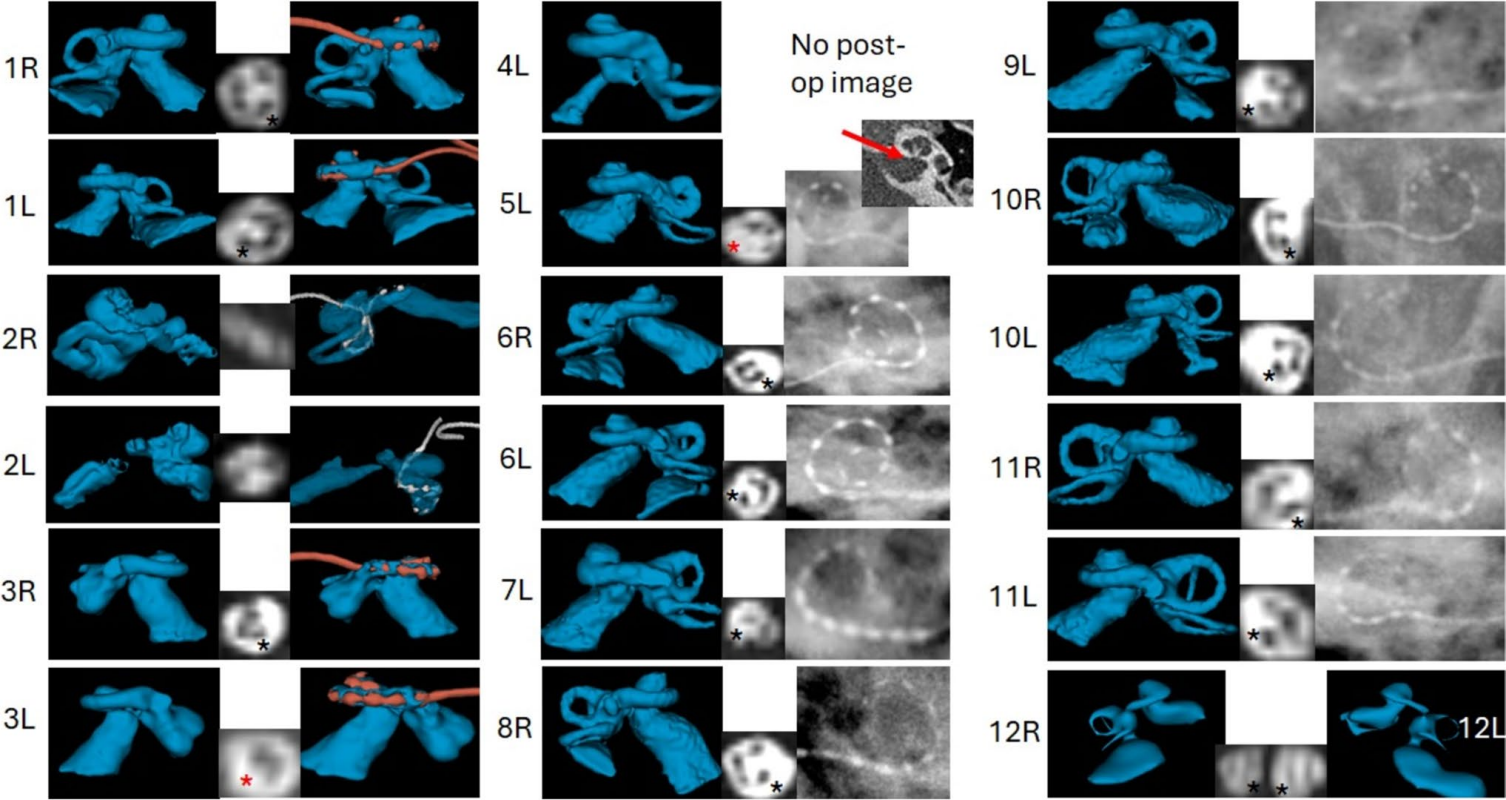
3D image of all ears implanted with CI in this cohortand red arrow in case 5L points to the cochlear aperture stenosis

Our database showed a total of 12 children radiologically diagnosed with inner ear malformation out of which 19 ears were treated with CI. All subjects were implanted with MED-EL CI device coupled with different electrode variants. Normal anatomy (NA) inner ear was seen in 2 ears (case 11), enlarged vestibular aqueduct (EVA) in 4 ears (cases 1L, 6R, 6L, and 8R) incomplete partition (IP) type II in 4 ears (1R, 9L, 12R, 12L), IP type I in 3 ears (7L, 10R, 10L), cochlear hypoplasia (CH) in 2 ears (3R, 3L), cochlear aperture stenosis or cochlear nerve deficiency (CND) in 2 ears (4L, 5L), and cavity type malformation in 2 ears (2R, 2L). MRI confirmed the presence of cochlear nerve in all subjects except 2R, 2L, and 3L. Subject 2 has common cavity type malformation in which we observed only 2 nerve bundles, one referring to facial and the other referring to both cochlea and vestibular portions of the inner ear. Hypoplastic cochlear nerve was seen in subject 5L pointed by red asterisk. MRI was not available for the ear 4L. Figure 2 shows 3D image of all ears implanted with CI in this cohort and red arrow in case 5L points to the cochlear aperture stenosis. Demographics of the study cohort are given in Table 1.

**Table 1:** Demographics of the cohort showing deafness onset, anatomical types, electrode variants, surgical observation and hearing outcomes with CI

Subject Nr	Deafness onset	Anatomical type		Observation of Cochlear nerve		Electrode variant		Surgical observation	PTA (dB) @ 6 months		SIR (time point)	
		R	L	R	L	R	L		R	L	R	L
1	Pre-lingual	IP II	EVA	yes	yes	FS	FS	Gusher	40	40	n/a	n/a
2	Pre-lingual	CC	CC	n/a	n/a	STD	STD	n/a	40	40	1 (3rd year)	1 (3rd year)
3	Pre-lingual	CH	CH	yes	no	F24	F24	Normal	45	80	n/a	n/a
4	Pre-lingual	-	CH/CND	-	n/a	-	F24	Anomaly of ossicles, absence of oval window	-	80	-	1 (3rd year)
5	Pre-lingual	-	Stenosis	-	hypoplastic	-	F28	normal	-	60	-	1 (3rd year)
6	Post-lingual	EVA	EVA	yes	yes	F28	F28	Gusher	30	30	4 (1st year)	4 (1st year)
7	Pre-lingual	-	IP I	-	yes	-	F20	Severe gusher	-	70	-	1 (3rd year)
8	Post-lingual	EVA	-	yes	-	F26	-	Gusher	40	n/a	4 (1st year)	-
9	Pre-lingual	-	IP II	-	yes	-	F24	Anomaly of ossicles and abnormal facial nerve course	-	40	-	2 (3rd year)
10	Pre-lingual	IP I	IP I	yes	yes	FO19	FO19	normal	40	40	3 (3rd year)	3 (3rd year)
11	Pre-lingual	NA	NA	yes	yes	F24	F24	Anomaly of round window	50	50	4 (3rd year)	4 (3rd year)
12	Pre-lingual	IP II	IP II	yes	yes	F28	F28	n/a	40	65	4 (3rd year)	4 (3rd year)

FS: FLEX SOFT (31.5 mm); STD: STANDARD (31.5 mm); F24: FLEX24 (24 mm); F26: FLEX26 (26 mm); F28: FLEX28 (28 mm); F20: FLEX20 (20 mm); FO19: FORM19 (20 mm); IP II: incomplete partition (IP) type II; EVA: enlarged vestibular aqueduct; CC: common cavity; CH: cochlear hypoplasia; CND: cochlear nerve deficiency. NA: normal anatomy: n/a: not available; PTA: pure tone average; SIR: speech intelligibility rating; n/a: image not available

### Intra-operative observations and post-operative electrode position

Gusher was observed in 4 cases (1, 6, 7, and 8) during the surgery, anomaly of middle ear ossicles was seen in 2 cases (4, 9) and RW was hidden in case number 11. Rest of the cases though had malformed inner ear anatomies did not show any surgical complications. Post-op CT was available for 6 ears whereas plain film x-ray was available for 10 ears and no post-op images were available for 3 ears. A 31.5 mm long straight electrode (FLEX SOFT, STANDARD) was fully implanted in cases with EVA, IP II, and common cavity. A 28 mm long electrode (FLEX28) was fully implanted in cases with IP II, EVA, and CH. A 26 mm long electrode (FLEX26) was partially implanted with 10 channels inside the cochlea in a case with EVA. A 24 mm long electrode (FLEX24) was fully implanted in cases with NA, CH, and CND. A 20 mm long electrode (FLEX20 and FORM19) were successfully implanted in cases with IP I, although FLEX20 in case 7L had partial insertion with basal most channel C12 is placed outside the cochlea. STANDARD electrode in case 2R and 2L (common cavity type malformation) is mainly placed in the posterior canal of the vestibular portion. Please refer to Table 1 and Fig. 2

### Post-operative hearing benefits with CI

A higher hearing threshold in PTA test was observed in cases 3L (CH), 5L (Stenosis), 7L (IP I), and 12L (IP II). SIR scores were 4 in the scale of 1–5 for NA and EVA type anatomies and between 1–3 for severe malformation types like CH, stenosis and IP. PTAs ≥ 60 dB was observed in cases of 3L (absent of cochlear nerve), 4L (cochlear nerve deficiency), 5L (cochlear aperture stenosis) and 7L (unilateral CI). Exceptionally the case 12L that has IP type II malformation along with the presence of cochlear nerve also showed PTA of 65 dB.

## Discussion

This is the first study to our limited knowledge from Europe to report on 3D images of inner ear from all subjects in the cohort diagnosed with inner ear malformation along with electrode position inside the cochlea in children. Also, the cross-section at the mid-length of internal auditory canal in MRI was checked following the procedure described by Assiri et al. [14] and reported the presence of cochlear nerve bundle. Though the study population is small yet different inner ear anatomies including normal anatomy, EVA, IP I, IP II, CH, CND/aperture stenosis and CC were seen in this study.

Surgical complication including CSF gusher was seen in anatomies of EVA and IP II and not in IP I and CC. Muscle tissue ring around the electrode was good enough to seal the cochlear entrance stopping the gusher in those cases. Later we realized that dedicated electrode like FORM19 (20 mm) and FORM24 (24 mm) from MED-EL electrode portfolio comes with a special cork type stopper at the basal end to seal the cochlear entrance with ease which will be considered in our future cases with expected CSF gusher. In this study, electrode choice was not made based on the inner ear anatomy rather based on the availability of device in the clinic. As a result, longer length flexible electrode was implanted in cases with cystic apex like (1R, 1L, 6R, 6L) and shorter length flexible electrode was implanted in a case with regular anatomy (11R, 11L). The one case of CC where the electrode was placed in the posterior canal of the vestibular portion was not the best electrode placement in this group.

As the CI field is moving towards the individualized CI treatment and CI companies offering different electrode variants [16], it is in the best interest of patients, we envision to choose electrodes based on individual’s cochlear size and anatomy in all our future cases. If this can further help patients to get additional benefit from CI is yet to be studied. Ozkan et al. has recently reported better CI outcomes in patients with less severe inner ear malformation types and vice versa [6] and the current study also observed this trend. Subjects with NA, EVA, and IP II had an SIR score of either 4 or 3, whereas subjects with CH, CC and CND had an SIR score of only 1. One case of IP I bilaterally implanted (C10, C11) had an SIR score of 3 whereas another case of IP I (7L) unilaterally implanted had SIR score of only 1. Presence of cochlear nerve bundle was confirmed in both these subjects with IP type I malformation type. Case 3 diagnosed with CH type malformation showed the presence of cochlear nerve bundle on the right side for which the PTA threshold was 40 dB whereas on the left side with absent cochlear nerve bundle, the PTA threshold was 80 dB. A detailed pre-operative image analysis looking at both CT and MRI is needed to understand the inner ear anatomy as well on the presence or absence of cochlear nerve. This can help to make a rough estimation on the hearing outcomes with CI.

Our future work will aim at studying electrically evoked auditory brainstem response (eABR) using the CI which will help to understand if the CI is stimulating the auditory cortex effectively or not.

### Limitations

One of the limitations of this study is the small number of subjects to make any conclusion on the anatomical type and hearing benefits with CI, although subjects with better developed inner ear anatomy score higher in the SIR compared to subjects with severely malformed inner ear anatomy. Moreover, we did not look at how many individual electrode channels out of 12 were contributing to hearing in severely malformed inner ear anatomies to bring meaningful benefit with CI.

## Conclusion

CI in general is beneficial in subjects with malformed inner ear anatomies. Severity of malformation have a negative effect on the hearing outcomes with CI. MRI is an important tool in the identification of cochlear nerve bundle in subjects diagnosed with malformed inner ear anatomies. Gusher of cochlear fluid was observed in less severe malformation types like EVA and IP II. Careful selection of CI electrode length matching cochlear size and anatomy is needed to minimize electrode placement complications and bring better benefit of CI device to patients.
